# Moral Injury in Elite Sport: Trauma in the Failure of the Athlete’s Quest

**DOI:** 10.1007/s10943-026-02574-w

**Published:** 2026-02-03

**Authors:** Matthew P. Hawkins

**Affiliations:** Theology Programme, University of Otago, Dunedin, New Zealand

**Keywords:** Elite sport, Chaplaincy, Moral injury, Integrity, Maltreatment, Wellbeing New Zealand

## Abstract

This article considers whether the trauma syndrome moral injury, as developed within the military context, is applicable to elite sport and to sport’s wellbeing and integrity discourse. In the face of mounting wellbeing and integrity problems faced within elite sport, the literature examining the wellbeing and integrity of sport, is calling for a multidisciplinary response that considers the moral and ethical frameworks within elite sports’ institutions. Moral injury presents as such a response. Examined is the growing use of moral injury in the civilian context and specifically amongst healthcare workers. Drawing together this work, moral injury is posited as applicable to elite sport though contextually contingent. As has been summarised within the military context for potentially morally injurious events, there is often the *taking of life*, whereas in the medical context it is in the *failure to save life.* This article argues that in elite sport the context for potentially morally injurious events is in the *failure of the quest*, where the quest is the elite athlete’s socially constructed ultimate existential purpose. Its traumatic loss can cause the shattering of identity and teleological meaning, and the loss of trust in self and others.

## Introduction

If sport is reduced to the cult of the human body, forgetting the primacy of the spirit or if it were to hinder your moral and intellectual development, or result in your serving less than noble aims, then it would lose its true significance and, in the long run, it would become even harmful to your healthy and full growth as human persons.

*Pope John Paul II, 2 September 1987 (Lixey et al., 2012, pp. 28, 29)*.

Renowned for his celebration of sport, the late Pope John Paul II’s words to the athletes of the 1987 Athletics World Championships in Rome are a pontifical plea warning of the peril to sport and its people by reducing sport to the veneration of human athletic performance. Forewarned is harm both to the integrity of sport itself and the wellbeing of people in sport. Such harm to individuals’ spirit, morality, mind, purpose, significance, and dignity is of deep concern to sports chaplains and others whose concern is for the wellbeing of people in sport.

This article considers whether moral injury, a trauma-based syndrome first identified within US military combat veterans from the war in Vietnam, is applicable to elite sport and to sport’s wellbeing and integrity discourse. My approach is to briefly examine moral injury as it has developed largely within the military context before discussing elite sport’s wellbeing and integrity problems. I then consider the rationale behind moral injury being applied to civilian populations, specifically healthcare workers, before taking a similar approach to applying moral injury in elite sport.

Sports chaplaincy is the provision of ongoing pastoral and spiritual care, by permission, to those of faith or no faith, for the holistic wellbeing of all involved in the community of sport. Most sports chaplains in New Zealand, where the author resides, and Australia are volunteers giving what discretionary time they can to a sport community. Unsurprisingly therefore amongst sports chaplains there is considerable variation across a training and qualification spectrum from informal to informal. Given this variation, sports chaplaincy can learn much from the discourse on moral injury in the military context and from military chaplains.

Elite sport is commonly defined by the level ascribed to its competition. Sport is ‘elite’ if it is professional, the Olympics, world championships, or intercollegiate (Aron et al., 2019, p. 779). In this article, ‘elite competition’ refers to such competitions and ‘elite sport’ denotes the institutions, people and systems that are oriented towards developing, coaching, and managing athletes competing in elite competitions.

## Moral Injury

Jonathan Shay used the phrase ‘moral injury’ in his 1994 book, *Achilles in Vietnam: Combat Trauma and the Undoing of Character*. Shay was reflecting on his professional experience of treating Vietnam War veterans with lasting painful emotions of guilt, shame, and anger that did not fit into the diagnosis and treatment of PTSD. In his sequel, *Odysseus in America: Combat Trauma and the Trials of Homecoming*, Shay defined moral injury as lasting harm from a betrayal of what is right by someone who holds legitimate authority in a high stakes situation (Shay, 2002, p. 240).

Instrumental in the development of moral injury was the seminal paper by Brett Litz and his team in 2009. They observed a second form of moral injury that arises from transgressive experiences that are potentially morally injurious and formally described potentially morally injurious events (PMIEs) as experiences where one perpetrates, fails to prevent, bears witness to, or learns about acts that transgress deeply held moral beliefs and expectations. The lasting harm is holistic—psychological, biological, spiritual, behavioural, and social (Litz et al., 2009, pp. 697, 700, 701).

Despite having similar sequelae, moral injury is not to be confused with PTSD (Litz et al., 2022, p. 2; Litz et al., 2009, pp. 697, 700, 701). PTSD is diagnosed when a severe traumatic fear-based stressor is present along with four major fear and trauma-based symptom clusters that adversely affect functioning in daily life (Koenig & Al Zaben, 2021, pp. 2994, 2995; Mattson et al., 2025, p. 301). In contrast, moral injury can develop when transgressions of moral beliefs are experienced with consequent feelings of guilt, shame, betrayal, anger, moral concern, difficulty forgiving, loss of meaning, loss of trust, self-condemnation, spiritual struggles, and loss of religious faith (Koenig & Al Zaben, 2021, pp. 2994, 2995; Mattson et al., 2025, p. 301). Therefore, moral injury represents a broader bio-psycho-socio-spiritual-behavioural syndrome independent from PTSD, though often comorbid with PTSD (Carey et al., 2025, p. 99; Sloane, 2023, p. 557).

### Interdisciplinary Approach

An underlying discourse tension exists within the phrase ‘moral injury.’ Arguably ‘moral’ belongs in the humanities whereas ‘injury’ sits in the medical domain. Where does moral injury belong? From the outset, Shay and Litz, military psychiatrists, acknowledge and welcome an interdisciplinary approach in researching moral injury, its interventions and preventions. Theirs is a ‘both-and’ approach. Shay’s definition invites the exploration of leadership paradigms and the ethical use of power as preventive of moral injury (Shay, 2002, pp. 205–242). Litz et al. (2009) express that the complexity of the subject matter requires the attention of expertise from within biology, philosophy, sociology and social psychology, law, religion, and mental health (Litz et al., 2009, p. 696). Shay remarks that religious and cultural therapies are not only possible but may well be superior to what mental health professionals conventionally offer (Shay, 2002, p. 152).

### The ‘Moral’ in Moral Injury

Litz et al. (2009) describe morals as the personal and shared familial, cultural, societal, and legal rules for social behaviour, either tacit or explicit. These are fundamental assumptions about how things ought to be and how one ought to behave in the world (Litz et al., 2009, p. 699). Medical ethicist Jonathan Cahill and his team (Cahill et al., 2023) argue that moral identity is formed in the context of communities and narratives that provide moral frameworks which situate the self and others relative to the good. Moral emotions serve to maintain a moral code and are driven by expectations of others’ responses to perceived transgressions. Emotions such as embarrassment, self-oriented pride, other-oriented gratitude, guilt, and shame shape moral behaviours (Cahill et al., 2023, pp. 225, 226).

Expanding this understanding, ethicist Aristotle Papanikolaou (2020) points towards virtue ethics and that the ‘moral’ in moral injury must have something to do with ‘being’ and not simply ‘doing’ (Papanikolaou, 2020, pp. 95, 96). Papanikolaou observes that being virtuous entails being for the good in a way that is persistently excellent. ‘Being for the good’ is defined as loving good, liking it, respecting it, desiring it, standing for it, speaking well of it, promoting it, protecting it, and being disposed to such things.

One can be virtuous and still breach social mores. As Jeremy Sabella (2019) describes, when National Football League (NFL) quarter-back Colin Kaepernick chose to ‘take a knee’ in 2016 during the pre-game playing of the USA’s national anthem, he breached social mores with a virtuous act. Kaepernick was protesting the continued discrimination and oppression of people of colour in the USA, an embodiment of values that transcend sport and nation (Sabella, 2019, pp. 456, 457). His actions drew sharp criticism. Against the backdrop of a society for whom pre-game corporate singing of the national anthem is elevated to a sacred ritual and ‘taking a knee’ is reserved for post-game prayers, Kaepernick was committing sacrilege (Sabella, 2019, p. 460).

### The ‘Injury’ in Moral Injury

Shay (2014) describes the injury as harm to character where one’s ideals, ambitions, and attachments begin to change and shrink. One’s capacity for trust is impaired and even destroyed. Despair, suicidality, and interpersonal violence is elevated (Shay, 2014, pp. 183–186).

As observed in the military by Litz et al. (2024), moral injury from violations of us-group (e.g. the unit) rules by self or others is characterised by intense moral emotions (guilt, shame, anger, disgust), threats to personal and shared identity (belongingness) and social bonds, and losses of faith in personal or collective humanity. They suggest that these unique outcomes stem from losses of valued activity or no longer being valued by others in a kinship group, which otherwise positively inform identity and sense of purpose, promote safety and are rewarding (Litz et al., 2024, p. 151).

According to Cahill et al. (2023), the painful emotions attached to moral injury are not pathological but are appropriate responses to perceived moral failures and transgressions by self or others. Importantly they say, moral injury is not a disorder in the clinical sense (Cahill et al., 2023, p. 227).

Moral philosopher Jay Bernstein defines moral injury as harm to dignity (Bernstein, 2005, 2015). Bernstein describes a person’s dignity as a social construction that comes to be through the forms of recognition which one attains in life’s journey from infancy to adult autonomy (Bernstein, 2005, p. 316). Dignity is the standing or status of persons considered as having intrinsic worth. Moral injury occurs when an individual works against another by violating the moral norms necessary for achieving adult autonomy. The victim experiences regression into a state of fragmentation and incompleteness (Bernstein, 2015, p. 1).

### Clinical Definition

A clinical definition of moral injury continues to be debated and developed (Koenig & Al Zaben, 2021, p. 2990). However, in one literature review (Hodgson & Carey, 2017), Timothy Hodgson and Lindsay Carey surmised that the most comprehensive and seemingly practical clinical definition is one formulated by Jeremy Jinkerson:

Phenomenologically, *moral injury* represents a particular trauma syndrome including psychological, existential, behavioural, and interpersonal issues that emerge following perceived violations of deep moral beliefs by oneself or trusted individuals (i.e., morally injurious experiences). These experiences cause significant moral dissonance, which if unresolved, leads to the development of its core symptoms: (a) guilt, (b) shame, (c) spiritual/existential conflict including subjective loss of meaning in life (or questioning of meaning in life), and (d) a loss of trust in self, others, and/or transcendental/ultimate beings. Its secondary symptomatic features include (a) depression, (b) anxiety, (c) anger, (d) re-experiencing of the moral conflict, (e) self-harm (i.e., suicidal ideation/behaviour, substance abuse, self-sabotage), and (f) social problems (e.g., social alienation, other interpersonal difficulty). It is likely that core symptomatic features influence the development of secondary symptomatic features. (Jinkerson, 2016, p. 126)

Jinkerson (2016) says that for moral injury to be identified there must be present a history of morally injurious event exposure, guilt, and at least two additional listed symptoms. Despite the thoroughness of Jinkerson’s definition, it does not expressly include ´betrayal´ (Hodgson & Carey, 2017, p. 1219) or ´suppression´ (International Centre for Moral Injury, 2025) alongside violation.

## Elite Sports’ Wellbeing and Integrity Problems

With gymnastics at the forefront, elite sport was put on notice in the wake of the 2016 revelations of sexual abuse within United States of America Gymnastics (USAG) by Dr Larry Nassar (McPhee & Dowden, 2018). Similar allegations of abuse within gymnastics surfaced around the world prompting several national gymnastics governing bodies to commission independent reviews. These included Switzerland (Rudin Cantieni, 2021), New Zealand (Howman et al., 2021), Australia (Australian Human Rights Commission, 2021), the United Kingdom (Whyte, 2022), the Netherlands (Royal Dutch Gymnastics Federation, 2021), and Canada (McLaren Global Sport Solutions Inc, 2023). These reports have similar findings of most gymnasts expressing positive experiences, but also toxic cultures and examples of abuse and maltreatment at all levels, with coaches, judges, and staff also reporting maltreatment. The Australian Human Rights Commission (AHRC) in its review determined that the key drivers of toxic culture within gymnastics were a ‘win-at-all-costs’ approach, the young age of female athletes, a culture of control, and a tolerance of negative behaviours while noting that several of *these key drivers are also evident in many other sports* (Australian Human Rights Commission, 2021, pp. 9, 28, 29) (emphasis added).

The AHRC’s findings are reflective of a broader systematic review of organisational factors and non-accidental violence in sport. Sports management scientists Victoria Roberts, Victor Sojo, and Felix Grant (2020) concluded that organisational tolerance for abuse and conformity to dominant values within sport consistently enabled and motivated psychological, physical and sexual abuse of athletes. Power imbalance and isolation enabled sexual abuse. Winner-take-all reward systems motivated physical abuse and the belief that non-accidental violence positively affected performance motivated psychological and physical abuse. They recommend a whole-of-system approach to the prevention and management of non-accidental violence in sport (Roberts et al., 2020, p. 25).

This finding would have come as no surprise to sports ethicist Mark Hamilton who observed in 2011 that much of the discussion in sports ethics concerns analysis of the actions of players and coaches regarding how the game is played, and topics including fair play, doping, gender issues, sport betting, fan misbehaviour, and issues of violence (Hamilton, 2011, p. 25). He goes on to say that we very often fail to question whether there might be something essentially wrong with sport itself.

In 2020, the journal *Sport Management Review* dedicated an entire edition to elite sport’s wellbeing issues. Editor Emma Kavanagh and her team advocated for integrity as a guiding principle in sport management research and practice with a focus on managing abuse in sport that does not settle for a ‘do no harm’ ethos. They argued that sports spaces and experiences should be safe, fair, positive, inclusive, and enriching—the absence of poor welfare, abuse, and violence is not sufficient (Kavanagh et al., 2020, p. 5). Picking up on this discussion and underlying my consideration of moral injury in this article is the paradigm that sports organisations have a moral duty of beneficence over and against the prevailing default of non-maleficence or ‘do no harm.’

## Moral Injury Applied to Civilian Populations

There has been growing interest in moral injury in civilian populations outside the military (Koenig & Al Zaben, 2021, p. 2997; Litz et al., 2022, p. 13). While moral injury has predominantly been studied in the military with clinical definitions, measures, and interventions developed in and for that context, recent consideration and research has expanded to include civilian populations. Some of these are: survivors of abuse (Andrews et al., 2024), public safety personnel (Roth et al., 2023), healthcare professionals in the COVID-19 pandemic response (Cahill et al., 2023; French et al., 2022; Rushton et al., 2021; Shale, 2020; Shibaoka, 2024; Sloane, 2023), youth (Chaplo et al., 2019; Kidwell & Kerig, 2023), emerging adults with child welfare histories (Haight et al., 2022; Soffer-Elnekave et al., 2023), social workers (Reamer, 2022), Japanese university students involved in sport (Toyoda et al., 2022), workplace bullying (Anderson & Mftc, 2021), and detained youth (Alexander et al., 2024).

With a purview across all society and nodding to the growing recognition of moral injury, the American Psychiatric Association (APA) recently added ‘Moral Problem’ to Z-code Z65.8 to add and connect moral problems, which include moral dilemmas, distress and injury, to the “Religious or Spiritual Problem” category in the DSM-V-TR (American Psychiatric Association (APA), 2025, p. 11; Mattson et al., 2025, pp. 297, 298). Moral problems include experiences that disrupt one’s understanding of right and wrong, or sense of goodness of oneself, others or institutions (American Psychiatric Association (APA), 2025, p. 11). Mattson et al. (2025) observe that ‘Moral Problem’ is sufficiently broad to include moral dilemmas, moral distress, and moral injury, in that the individual’s moral identity is transgressed, questioned, challenged, or outright contradicted, leading to varying levels of distress most often in the form of guilt, shame, and blame towards self and others (Mattson et al., 2025, p. 299).

However, caution has been voiced questioning moral injury’s applicability to civilian contexts. Unique to the military context are the required extreme actions within war that can violate the very basis of one’s moral identity which are often violent and/or traumatic (Nakashima Brock & Lettini, 2012, p. 52; Sloane, 2023, p. 556; Wiinikka-Lydon, 2019, p. 176). The caution is against bringing the literature on moral injury and associated measures from the military context to bear on understandings of and responses to putative moral injury in civilian contexts without careful consideration (Sloane, 2023, pp. 556–558). Further risked is the dilution of moral injury’s power to name and describe the deep harm caused to combat veterans who have experienced moral trauma (Shibaoka, 2024, p. 5).

### Medical Moral Injury

Caution noted, Andrew Sloane argues that the COVID-19 pandemic made us aware of medical moral injury (Sloane, 2023). His argument first distinguishes the medical context from the military context and considers what effect this will have on presenting sequelae. Sloane observes that war has a near continuous presence of threatened physical violence and trauma, which participants in war often experience as extreme threat to self, bodily integrity, or life itself. This is not true of the medical context where trauma and violence are not commonly present, though not unheard of. For example, PTSD is a likely comorbidity with military moral injury, but it is unlikely in the medical context unless the healthcare workers are first responders (Sloane, 2023, pp. 560, 561). Therefore, Sloane argues that in the medical context moral injury is more likely to be chronic than acute (Sloane, 2023, pp. 563, 564).

Sloanes’ argument in describing healthcare environments parallels Shay’s (1994) description of armies as social constructions with shared expectations and values that form a moral world, and as world-making institutions creating social power. Where social power is created and exists, then there is a social contract where the powerholder is a fiduciary and is assumed to act in fairness and in the best interests of those over whom social power is exercised—this is ‘what is right’ (Shay, 1994, pp. 3–19)*.*

Sloane (2023) observes that like military personnel, healthcare workers are highly morally motivated with initial moral impulses shaped by moral codes learnt and entrenched in their training and practice (Sloane, 2023, p. 558). He goes on to describe that during the COVID-19 pandemic healthcare workers experienced betrayal, violation and/or suppression in high stakes situations concerning patient wellbeing (Sloane, 2023, pp. 558, 559). Citing the nursing literature on moral distress, Sloane notes the similarities with military moral injury making a few observations. First, not all dilemmas are distressing, and not all moral distress leaves a moral residue. Second, nursing literature reflects the same two currents present in military moral injury literature: PMIEs as the result of failures or abuses of power, systems or authorities, and of one’s own actions. Third, significant and *repeated* moral distress results in *damage* and *dysfunction*, not just discomfort (Sloane, 2023, p. 563).

Sloane’s conclusion is that moral injury is applicable to medical contexts, albeit the causes and particular patterns of damage will generally differ from those of military moral injury, and medical moral injury is more likely to be chronic than acute. This is significant because differentiating kinds of moral injury and the contexts in which they occur matters to diagnosis and effective clinical, moral, and theological responses (Sloane, 2023, p. 564). Atsushi Shibaoka in a later article helpfully summarised that the military context for PMIEs is often the *taking of life*, whereas in the medical context the PMIE is in the *failure to save life* (Shibaoka, 2024, pp. 6, 7).

## Moral Injury Applied to Elite Sport

If the military context for PMIEs is often the *taking of life* and the medical context for PMIEs is in the *failure to save life,* then I suggest that the elite sport context for PMIEs is the *failure of the quest* where the quest is the elite athlete’s socially constructed ultimate existential purpose and telos in their sport. As will be discussed below, elite athletes strive to be the best that they can be in their sport in whatever form that takes. Invariably this striving becomes socially constructed due to social complexities within elite sport. Athletes’ identities are shaped and sometimes shattered by elite sport’s institutional power and demands.

Moral injury does not appear in sport’s wellbeing literature. However, at the time of writing there are two papers associating moral injury with sport. The first (Atuel et al., 2021) argued for advancing a broader theoretical foundation for moral injury that has wider applicability into varieties of human experience beyond the military. The second is a Japanese study that investigated the mediating effect of moral injury on interpersonal violence victimisation in Japanese university athletes (Toyoda et al., 2022).

In both papers, the applicability of moral injury to sport is assumed with no consideration given to the influence of the contextual differences between the military and elite sport. The obvious risks are misappropriation and misapplication of moral injury as developed in the military context. However, the questions remain, ‘Is moral injury applicable to elite sport? And if so, how?’ I will endeavour to address these questions by treading in Shay’s and Sloane’s steps.

### Contextual Consideration

Sloane’s starting point is to compare and distinguish contexts. As in the medical context, the near continuous presence of threatened physical violence and trauma witnessed in wartime is also not true for elite sport. Equally, the theatre of war, though with rules of engagement, is a comparatively unadjudicated space against elite sport’s immediate administration of game rules by match officials. Cheating and match-fixing notwithstanding, unlike the theatre of war, the heavily officiated theatre of elite sport is not where one looks for potentially morally injurious events. Rather, attention is turned towards elite sport’s institutions, people and systems.

### Elite Sport’s High Stakes

While elite sport does not share the literal life or death high stakes ever present in the military, the high stakes that elite sport does share are identity, both personal and shared, and social bonds. The athlete’s quest lies at the centre. Traumatic loss threatens such and is one that disrupts a person’s sense of safety and control and causes the loss of a sense of identity and purpose (Prigerson et al., 1997, p. 1007).

Pinnacle events such as the Olympic Games and world championships give nations the opportunity for their athletes to perform on the global stage and in so doing develop national identity, pride, and further national interests (Ellis, 2021, p. 40). Using the Summer Olympic Games as an example, unsurprisingly the global super-powers of the USA, China and Russia hold the top three spots on the medal tables (Globan & Rewilak, 2024, p. 12). Akin to an international arms race, increasingly nations such as Great Britain, who have targeted cycling, are adopting strategic approaches to develop medal winning strategies at the Olympic games (Globan & Rewilak, 2024, p. 12).

In New Zealand elite sport has been described as the second most formative force in its evolving identity on the world stage (Warren, 2018, pp. 182, 183, 187). In the summer Olympics per capita New Zealand is ranked the fourth most successful nation (Globan & Rewilak, 2024, p. 12). New Zealand’s high-performance sport expresses the unequivocal expectation of return on investment into high-performance programmes (Heron et al., 2022, p. 6).

Failure by elite athletes often brings the public ire of media and pundits, and sometimes their administration. However, as will be discussed below in the case study on football academies, more devasting is the traumatic end of the quest that shatters the athlete’s socially constructed ultimate existential purpose and telos. Symbolising this loss are the termination of contracts, support systems, sponsorships, access to the club, training facilities and coaches, and the handing back of kit and sponsors' perks.

### Elite Sports as Social Constructions

Shay’s description of armies as social constructions (Shay, 1994, p. 6) also rings true for elite sport. Elite sports have shared expectations and values that can be embodied in formal regulations and authorities, and/or exist in circulated traditions and together form the moral world that most of the participants for most of the time consider as legitimate, natural and binding.

One example is rugby league in Australia where team environments have been described as ‘homosocial’ with codes of mate-ship and loyalty in tightly knit groups that are valuable for teamwork. But such environments but can also intensify toxicity such as sexism and group loyalty to override personal integrity (Packard Hill & Fuller, 2018, pp. 42, 43). Homosocial bonds between players and ex-players never cease to have social worth and these bonds can prevent players from speaking out against teammate’s violence and instances of abuse (Packard Hill & Fuller, 2018, p. 54).

Peter Harmer (1991) described the athlete-authority relationship in terms of mutual duty and obligation. In effect there exists a morally powerful implied social contract where the athlete in their quest is expected and required to perform. Therefore s/he promises to strive for excellence while the powerholder or authority promises to support him/her to the greatest extent possible in his/her struggle for excellence (Harmer, 1991, p. 27). The athlete’s quest is at the core and is most often the high stake.

According to Harmer (1991), it is a myth that ultimate performance can be achieved through the athlete’s innate talent, desire and training alone. The entourage of coaches, technical specialists, medical staff, and others around elite athletes is more telling of the truth. Elite sport is undeniably socio-political, and ultimate performance is achieved through a multiplicity of factors, many of which are outside the control of the athlete (Harmer, 1991, p. 32).

Another myth that sports sociologist Jay Coakley identifies within society is an unshakeable belief about the inherent purity and goodness of sport that he calls ‘the Great Sport Myth’ (Coakley, 2015, p. 403). He contends that many people combine this belief with two others: the purity and goodness of sport is transmitted to those who participate in or consume it, and sport inevitably leads to individual and community development (Coakley, 2015, p. 403). Undeniably exercise, physical fitness, and participation in sport and recreation have positive benefits physically, mentally, and socially, but academic research and discourse also reveals that serious harm can come from involvement in sport unless interventions are employed. Indeed, research in the USA shows that sport negatively affects athletes’ abilities to reason about moral issues and dilemmas in sport, and does not appear to cognitively develop young people (Beller & Stoll, 1995; Beller et al., 2004; Coakley, 2011; Rudd, 2008; Stoll et al., 2018).

### Elite Sport as World-Making Institutions

Like armies, elite sports are world-making institutions creating social power through institutionalising forces that diminish the athlete’s agency, subjugate athletes through a variety of vulnerabilities, and forging athlete identities centred on their sport and quest.

An athlete’s participation in elite sport is arguably volitional whereas a soldier’s participation in war often is not. However, most elite athletes start their quest in childhood or adolescence, becoming fixated on a sport and imagining themselves playing professionally, being a world or Olympic champion. As described by Kavanagh (2014), coerced consent begins early in the athlete’s inculcation into their sport (Kavanagh, 2014, p. 140):

Early in the involvement, the athlete becomes ‘converted’ to the worldview of the sporting subculture and becomes ‘entangled’ relatively quickly in a subtle but ever-increasing series of ‘commitments and obligations’. The further the athlete moves along the career path, the more commitments and obligations s/he has to make and meet, and the more entangled s/he also becomes in relationships with others in the sport. Finally, to the extent that reputations and identities are built and are seen as desirable, the athlete becomes increasingly committed and tied to his/her athletic career and continues along the performance pathway.

The social power differential between athletes and their governing institution exposes elite athletes to multiple vulnerabilities throughout their quest (Heron et al., 2022, pp. 23, 24). Centralisation into training hubs dislocate athletes from their established support networks and often require sacrificing life-balance bringing elements such as where to live, having a partner, employment and outside careers, having children, and attending events such as weddings, funerals, and birthdays. Most elite athletes are young and exposed to continual selection pressures compared to other stakeholders in the system.

Elite athletes are often dependent on the permission of their national sports organisation to compete internationally while their incomes are often contingent upon maintaining or improving placings in such competitions. The lack of legal employee rights and protections and the high costs of living, training and competing mean many elite athletes remain financially dependent on their family. Perhaps most telling, the athlete is the only person who can deliver performance and medals, but also generally has the least control over organisational structures and systems that directly affect them.

Selection and deselection processes often reveal the social power differential in elite sport. Decisions that lack transparency, are unexpected, ambiguous, prolonged, or inconsistent with stated guidelines and comparative selection decisions, cause elite athletes considerable stress (Brand et al., 2013; Fletcher & Hanton, 2003; McKenna & Thomas, 2007). Akin to traumatic loss by military personnel who are no longer valued by others in a kinship group as described by Litz et al. (2024), the harmful experiences of deselection are well documented and include psychological distress, emotions such as shock, brokenness, devastation and hurt, loss of identity, loss of friendships and career termination (Brand et al., 2013, pp. 118, 119; Brown & Potrac, 2009; Fletcher & Hanton, 2003; Neely & Dugdale, 2023; Roberts & Faull, 2013; Slade, 2024).

### Institutionalising Forces and Athlete Identity

Similar to the military, elite sports have institutionalising forces that can develop within athletes a mindset where sport *is* life and to lose it, or to be unable to play for whatever reason, can have catastrophic consequences for their sense of identity and welfare (Watson, 2019, p. 14). In the athlete’s quest, identity becomes an increasingly high stake to not only the athlete but to parents and caregivers who have entrusted their child, adolescent, or emerging adult to the care of coaches, managers, agents, and organisations who promote and recruit for sports academies and high-performance programmes.

During the developmentally important years of emerging adulthood between 18 and 25 it is common for elite athletes to become inculcated to his or her athletic career. In the general population there are five developmental features most prominently experienced during emerging adulthood. These are (1) identity exploration leading to subsequent identity choices, (2) instability due to identity explorations and associate experiences, (3) self-focus where knowledge, skills, and self-understanding are developed for later adult life, (4) feeling in-between with neither fixed adolescent or proper adulthood identities, and (5) possibilities/optimism about entering adult life and a sense of positivity about what lies ahead (Rice et al., 2022, p. 1).

However, in what has been described as athlete identity foreclosure (Brewer & Petitpas, 2017) young elite athletes come to see themselves solely as an athlete in a particular sport. Committed to becoming, or being an elite athlete, they avoid exploratory behaviour pertaining to other roles (Brewer & Petitpas, 2017, pp. 120, 121; Stewart et al., 2024, p. 1). This avoidance hinders the process of establishing a sense of self-identity, coping strategies, and confidence in their abilities to be successful in adult life (Brewer & Petitpas, 2017, pp. 118, 119). Should the quest falter, founder, or become wrecked, then the athlete’s whole sense of identity can be at risk (Slade, 2024, pp. 117–119; Sport New Zealand, 2019, p. 44; Watson, 2019, p. 15).

Sabrina Heimler’s research on the retirement transition of professional rugby union players in the United Kingdom (Heimler, 2022) showed that professional rugby players developed very strong athletic rugby identities with a focus on playing rugby above all else. They valued paradigms of going into battle and sacrifice for the team. However, such environments limited or provided no engagement with support that could help players transition from rugby’s institutions and their continued singular rugby identities (Heimler, 2022, pp. I, II).

### Betrayal, Violation, and/or Suppression

Where there is betrayal, violation, or suppression of deeply held or shared moral beliefs, then there is the potential for moral injury. In elite sport this can take the form of maltreatment. Maltreatment is becoming well documented and can be emotional abuse, physical abuse, forced physical exertion, neglect (rejecting, isolating, excluding), bullying, discrimination, and organisational maltreatment (Fortier et al., 2019, p. 1; Kavanagh et al., 2023, p. 2). Kavanagh identifies the coach-athlete relationship and peer-to-peer relationships as the primary relationships in which maltreatment occurred (Kavanagh, 2014, p. 147). The top four groups of athletes with a higher risk of maltreatment in sport are elite athletes, child athletes, athletes with a disability, and athletes who identify as LGBTQ (Mountjoy & Edwards, 2022, p. 157).

The sexual abuse of athletes by Nassar furnishes examples of personal and institutional betrayal, violation, and suppression. The independent investigation reported by McPhee and Dowden (2018) found that Nassar committed thousands of sexual assaults of athletes between the early 1990s and 2016. Nassar established himself as a gymnastics expert and trusted doctor in the early 1990s and became the USA Women’s National Team physician at the 1996 Olympic Games, a position that he held for nearly 20 years. It was under the guise of legitimate medical treatments that Nassar committed almost all his crimes. Misled by Nassar, many athletes said that while feeling discomfort, they did not think he was molesting them. Indeed, Nassar abused athletes when their parents were present by positioning his body between his actions and the sight of the parent (McPhee & Dowden, 2018, pp. 1–9).

McPhee and Dowden (2018) reported that Nassar acted within a system that facilitated his criminal acts. Institutions such as Michigan State University, USAG, the United States Olympic Committee, and individuals within these institutions enabled his abuse, did not treat complaints appropriately, and failed to stop him. Despite branding itself as a leader in athlete protection, USAG repeatedly failed to respond adequately to concrete reports of specific misconduct and instead imposed procedural requirements that blocked or delayed effective action (McPhee & Dowden, 2018, pp. 1–9).

The victim impact statements at Nassar’s trial expose guilt, shame, anger, disgust, loss of trust, and other sequelae anticipated in moral injury. Reported by McPhee and Dowden (2018), abuse survivors described grappling with self-doubt and self-blame as later in life they tried to figure out how they became so brainwashed as to not recognise the abuse at the time. Many speak of lifelong troubles with trust and intimacy. Others describe periods of anxiety, bouts of panic attacks, and paralysing flashbacks. Survivors explained how unwelcome memories invade at the most inopportune times and living with daily experiences of psychological and emotional pain. Many detailed how their lives spiralled out of control. The victims suffered from eating disorders and anxiety attacks. They describe long sleepless nights punctuated with nightmares that included images of being trapped in Nassar’s examination room that cause them to wake and vomit. Survivors described self-harm and thoughts of suicide (McPhee & Dowden, 2018, p. 155). Perhaps indicative of the moral distress experienced by parents, of the three suicides attributed to the aftermath of Nassar’s abuse, two were fathers of abuse victims (Mountjoy & Edwards, 2022, p. 158).

### Moral Injury in Elite Sport

Summarising the above discussion, though elite sport is contextually different to the military what is apparent is that the potential for morally injurious events is evident in elite sport. Also evident is the lasting harm and sequelae also associated with moral injury. This points towards an affirmative, though contingent, answer to the question, ‘Is moral injury applicable to elite sport?’ Different to military moral injury, where PTSD is often a comorbidity, moral injury in elite sport is less likely to have PTSD comorbidity. It is also anticipated that moral injury sequelae in elite sport will present as mild and chronic, with guilt being less prominent, while shame, anger, disgust, contempt, self-harm, social problems, existential-ontological conflict including subjective loss of meaning in life and identity, and a loss of trust in self, others, and/or faith being more salient.

### Application to Wellbeing and Integrity Discourse

If indeed moral injury finds contingent application in elite sport, how might moral injury’s nascent interdisciplinary discourse and research be applicable to wellbeing and integrity in elite sport? I suggest that moral injury provides a critical lens for sport’s ethicists, sociologists, management scientists, lawyers, and theologians while also assisting clinicians and chaplains to diagnose and treat people within elite sport whose lasting harms are caused by the betrayal, violation or suppression of deeply held or shared moral values.

Undoubtedly, clinicians will want further research, definitional clarity, applicability and treatments for moral injury in elite sports. In part, clinical discussion is moving towards what appears to be moral injury’s overlay with complex post-traumatic stress disorder (C-PTSD) as described in the WHO-ICD-11 (Maercker et al., 2022, p. 60) and moral distress described in nursing literature. However, it is beyond the scope of this article to consider a clinical research project for moral injury in elite sport. As noted above, clinical research work in moral injury has grown in momentum in civilian populations that can inform possible research projects. Importantly, Shibaoka cautions against using slightly amended military moral injury assessment tools on civilian populations whose contexts are markedly different to that of the military (Shibaoka, 2024, pp. 6, 7).

While Mark Hamilton observed that we very often fail to question whether there might be something essentially wrong with sport itself (Hamilton, 2011), that question is not without discussion within different disciplines. Theologians whose interest is elite sport note the serious wellbeing issues evident in elite sport and seek to provide spiritual and religious insight into these issues along with interventions (Ellis, 2014; Hoffman, 2010; Johnston, 1997; Lixey et al., 2012; Watson, 2019; White, 2011). Sports ethicists express concern that elite sport has a degrading influence on social mores and moral sensibilities (Beller & Stoll, 1995; Beller et al., 2004; Berg, 2018; Coakley, 2015; Stoll et al., 2018). Sociologists question sports influence to positively develop youth and the social structures within sport that open coaches and athletes to abusive relationships and non-accidental forms of violence (Coakley, 2011; Gaedicke et al., 2021; Ohlert et al., 2021; Parent & Fortier, 2018; Parent et al., 2022). Sports management scientists are actively seeking how to manage abuse within sport and promote integrity (Kavanagh et al., 2020). However, as wellbeing and integrity in elite sport has yet to be considered through the lens of moral injury a case study is now proffered albeit in brief.

### UK Football Academies as a Case Study for Moral Injury in Elite Sport

There is little doubt that football academies are developmental pathways into professional football but how do they affect players’ wellbeing through the lens of moral injury?

The European Club Association, 2012 report on youth football academies positioned them at the centre of the future for European club football (European Club Association, 2012). In the same legal jurisdiction, Articles 9 and 17 under the European Social Charter (Revised) (ESC-Rev) guarantee the provision of services that will assist in solving problems related to occupational choice and progress, and the protection of children and young persons against negligence, violence or exploitation. However, indicative of a non-maleficent posture, the ECA’s report is silent on deselection rates, deselection processes, transition support for released players, and footballing academies’ ESC-Rev obligations to deselected and released players.

Player churn is high in the UK’s football academies where approximately 2 per cent of 16-year-olds awarded academy scholarships are still playing in the UK’s top five footballing tiers at age 18 (Calvin, 2017, p. 4; McGlinchey et al., 2022, p. 1). These authors report that only 8 out of 400 players given a English Premier League (EPL) contract at 18 remained at the highest level by their twenty-second birthday. The implication is that almost all academy players will experience the failure and termination of their quest during their emerging adult years when they are most vulnerable to athlete identity foreclosure.

Thomas McGlinchey and his team’s study of elite youth players’ experiences of release from professional football academies in the UK found that youth players experienced marginalisation, being ‘left out in the cold’, emotional trauma in the contract-end meeting, and no club aftercare. Post release, players experienced feelings of depression, identity crisis and confusion, loss of self-worth, sadness, pain, anguish, anger, isolation, betrayal, being dehumanised, and personal worthlessness (McGlinchey et al., 2022, pp. 11, 12). Though about the experience of deselected football academy players, this study resonates with Litz et al’s. (2024) observations of military personnel who experienced traumatic loss and moral injury resulting in guilt, shame, anger, disgust, threats to personal and shared identity (belongingness) and social bonds, and losses of faith in personal or collective humanity (Litz et al., 2024, p. 151).

Employing Bernstein’s (2005, 2015) moral injury as harm to dignity, the described painful experiences can be explained as originating from the academy institution and staff who, having heavily influenced players’ worlds and footballing identity from childhood, withdraw their recognition of players’ potential at the very point of transition into footballing ‘adulthood’. The players’ experience of deselection and release is one of violation or betrayal of moral norms that affirm and protect one’s intrinsic worth. The players’ painful experiences are symptomatic of existential and ontological regression into states of fragmentation and incompleteness. Where there has been maltreatment and/or deselection and release processes that lack transparency the experience of violation, betrayal and/or suppression may be heightened. More acute is the experienced devastation to identity, dignity, beliefs, physical wellbeing, relationships, faith, and emotional and psychological development.

In my opinion McGlinchey et al’s. (2022) findings strongly suggest that the academy system is itself potentially morally injurious. However, care must be taken to not weaponize moral injury against academy staff who in their commission of harm may also be victims of a potentially morally injurious system. Through the lens of moral injury as harm to moral imagination (Wiinikka-Lydon, 2020), it is conceivable that the institutions and structures of football that systemically and politically filter relationships greatly narrow the ability of academy staff to creatively picture alternative relationships and futures. This harm to imagination funnels and screens how one evaluates others, how one creates meaning, what one hopes for, what one can hope for, and how one determines who is within one’s circle of concern. While avoiding weaponizing moral injury, recognising the perpetrator’s own moral injury is not cause for excuse or recuse of culpability. To do so only continues the perpetrator’s injury to others by failing to take due responsibility for one’s actions.

Given the world-making power and institutional hold that football academies have over the children and youth that are in them, the contextual conditions for moral injury are salient and require intentionality in benevolent management and leadership. Along these lines in the UK and against the backdrop of athlete maltreatment, is King’s Counsel Baroness Tanni Grey-Thompson’s, 2017 report to the UK Government (Grey-Thompson, 2017) recommending the formal establishment of ‘Duty of Care’ to athletes and participants in sport, to ensure welfare and safety are given deserved priority. Grey-Thompson’s definition of ‘Duty of Care’ is deliberately broad covering nine ‘themes’ of education, transition, representation of the participant’s voice, equality, diversity and inclusion, safeguarding, mental welfare, and safety, injury and medical issues (Grey-Thompson, 2017, pp. 3, 4).

Sports chaplains placed within these academies can advocate for greater benevolence in management and leadership. Academy staff themselves may also experience moral distress and injury within academy institutional structures. Yet, through turning from a posture of non-maleficence to benevolence much of this risk of moral harm can be mitigated and possibly eliminated. Sports chaplains have an important advocacy role supporting at the very least an intentional systemic shift to a duty of care as described within Grey-Thompson’s report as well as discharging the obligations in Articles 9 and 17 of the ESC-Rev.

## Conclusion

Elite sport’s wellbeing and integrity literature rarely frames as moral harm the maltreatment, non-accidental violence, and the various forms of abuse experienced by elite athletes. Yet even the concepts of wellbeing and integrity are contingent upon codes of morality and ethics that are both expressed and implied. What has become evident is that a non-maleficent stance of ‘do no harm’ is inadequate, rather the call is towards intentional benevolence. From the military context, the trauma syndrome described as moral injury, if applied with considered caution, is argued to also be an empowering concept in sport’s wellbeing and integrity discourse.

If the military context for PMIEs is often the *taking of life,* then I suggest that the elite sport context for PMIEs is the *failure of the quest* where the quest is the elite athlete’s socially constructed ultimate existential purpose and telos in their sport. Moral injury sequelae in elite sport will likely present as mild and chronic, with guilt being less prominent, while shame, anger, disgust, contempt, self-harm, social problems, existential-ontological conflict including subjective loss of meaning in life and identity, and a loss of trust in self, others, and/or faith being more salient.

Requiring interdisciplinary engagement, the subject area invites the exploration of leadership paradigms and the ethical use of power as preventive of moral injury as well as research and interventions from clinicians and care-providers whose concern are those whose wellbeing has been shattered as described by moral injury. Military chaplaincy’s growing body of moral injury literature is a valuable resource to sports chaplains for further study. Sports chaplaincy can learn much from military chaplaincy about moral injury. While the considerable literature on military moral injury and the developing resources from other civilian domains is undoubtedly a strong starting point, nevertheless careful contextual consideration is required when bringing such discourse, research, and study to bear on the domain of elite sport.
